# Supplementary figures and images for: A comprehensive SARS-CoV-2 genomic analysis identifies potential targets for drug repurposing
Source: PLoS One. 2021 Mar 18;16(3):e0248553. doi: 10.1371/journal.pone.0248553 (PMC7971693; doi:10.1371/journal.pone.0248553)

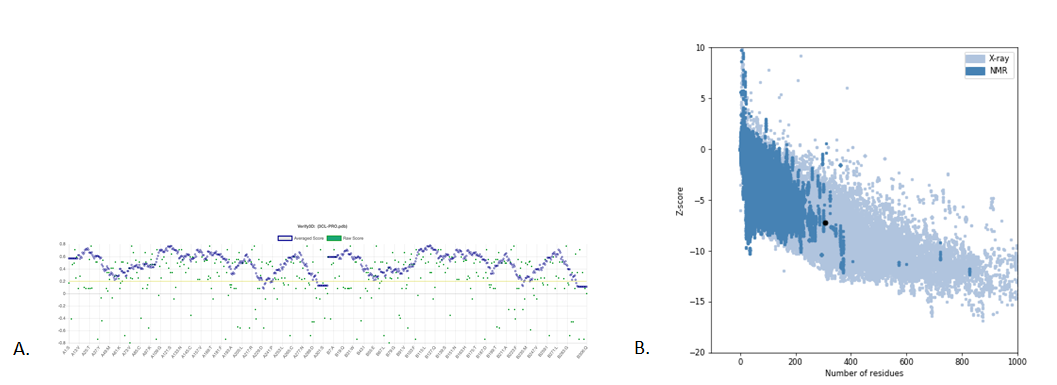

Supplement: S1 Fig — A. Validation of structure by ProSa, which compares the predicted model of 3CLpro (black dot), with a non-redundant set of crystallographic structures (light blue dots) and NMR structures (dark blue dots) and provides a Z-score. B. Validation of structure by Verify3D, which highlights the 3D-1D score for every atom of the predicted model. 94.28% of the residues in the 3CLpro model had a compatibility score of 0.2 or higher, which indicates a high quality structure. (TIF) [file pone.0248553.s001.tif]

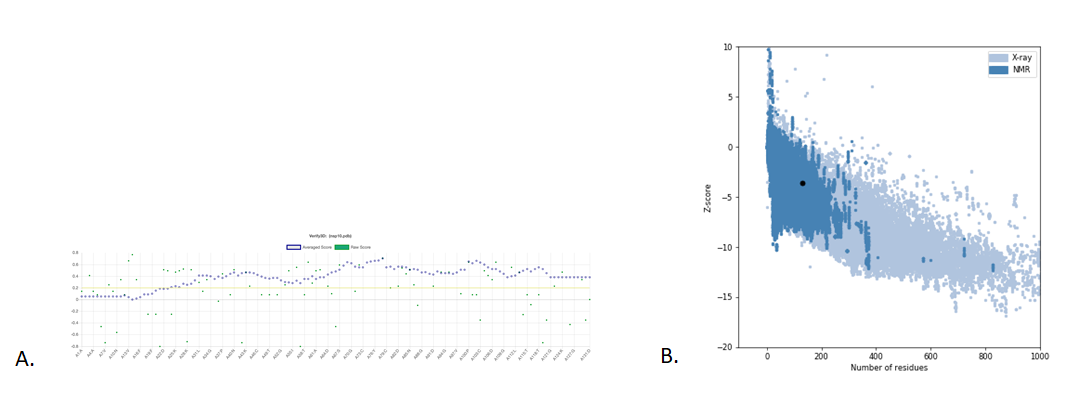

Supplement: S2 Fig — A. Validation of structure by ProSa, which compares the predicted model of 3CLpro (black dot), with a non-redundant set of crystallographic structures (light blue dots) and NMR structures (dark blue dots) and provides a Z-score B. Validation of structure by Verify3D, which highlights the 3D-1D score for every atom of the predicted model. 82.44% of the residues in the NSP10 model had a compatibility score of 0.2 or higher, which indicates a high-quality structure. (TIF) [file pone.0248553.s002.tif]

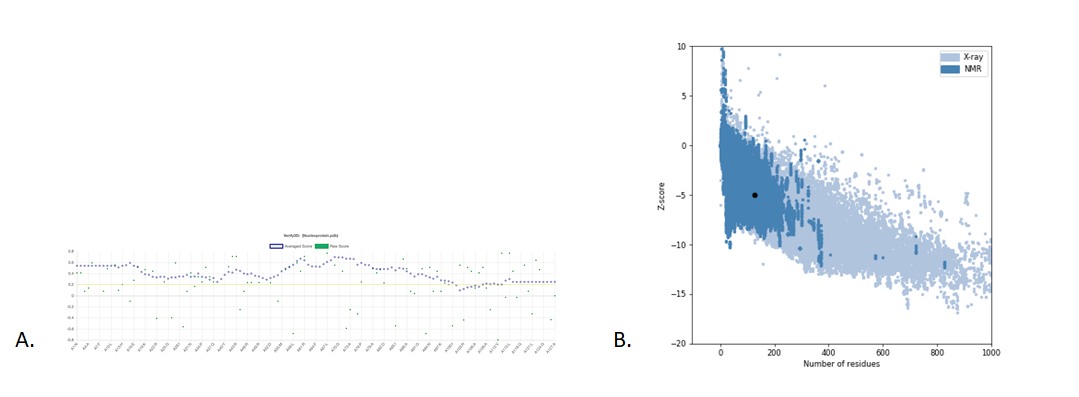

Supplement: S3 Fig — A. Validation of structure by ProSa, which compares the predicted model of 3CLpro (black dot), with a non-redundant set of crystallographic structures (light blue dots) and NMR structures (dark blue dots) and provides a Z-score.B. Validation of structure by Verify3D, which highlights the 3D-1D score for every atom of the predicted model. 94.49% of the residues in the nucleoprotein model had a compatibility score of 0.2 or higher, which indicates a high-quality structure. (TIF) [file pone.0248553.s003.tif]

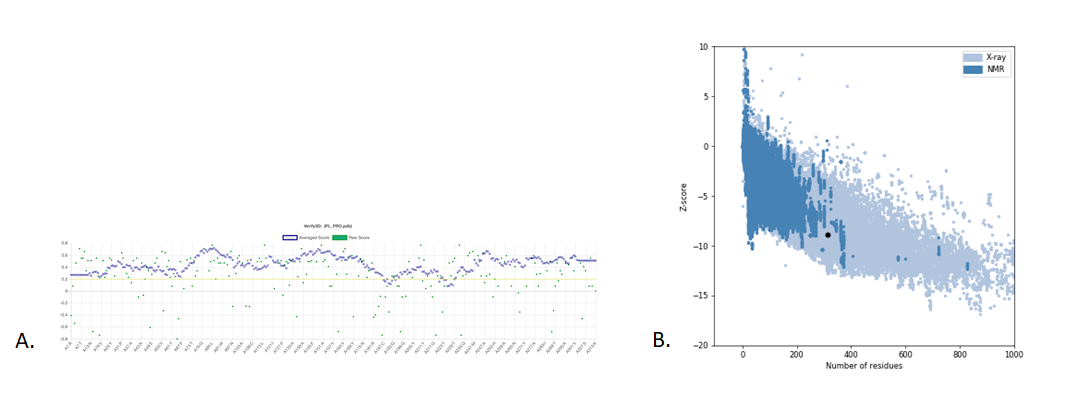

Supplement: S4 Fig — A. Validation of structure by ProSa, which compares the predicted model of 3CLpro (black dot), with a non-redundant set of crystallographic structures (light blue dots) and NMR structures (dark blue dots) and provides a Z-score.B. Validation of structure by Verify3D, which highlights the 3D-1D score for every atom of the predicted model. 95.85% of the residues in the PLpro model had a compatibility score of 0.2 or higher, which indicates a high-quality structure. (TIF) [file pone.0248553.s004.tif]

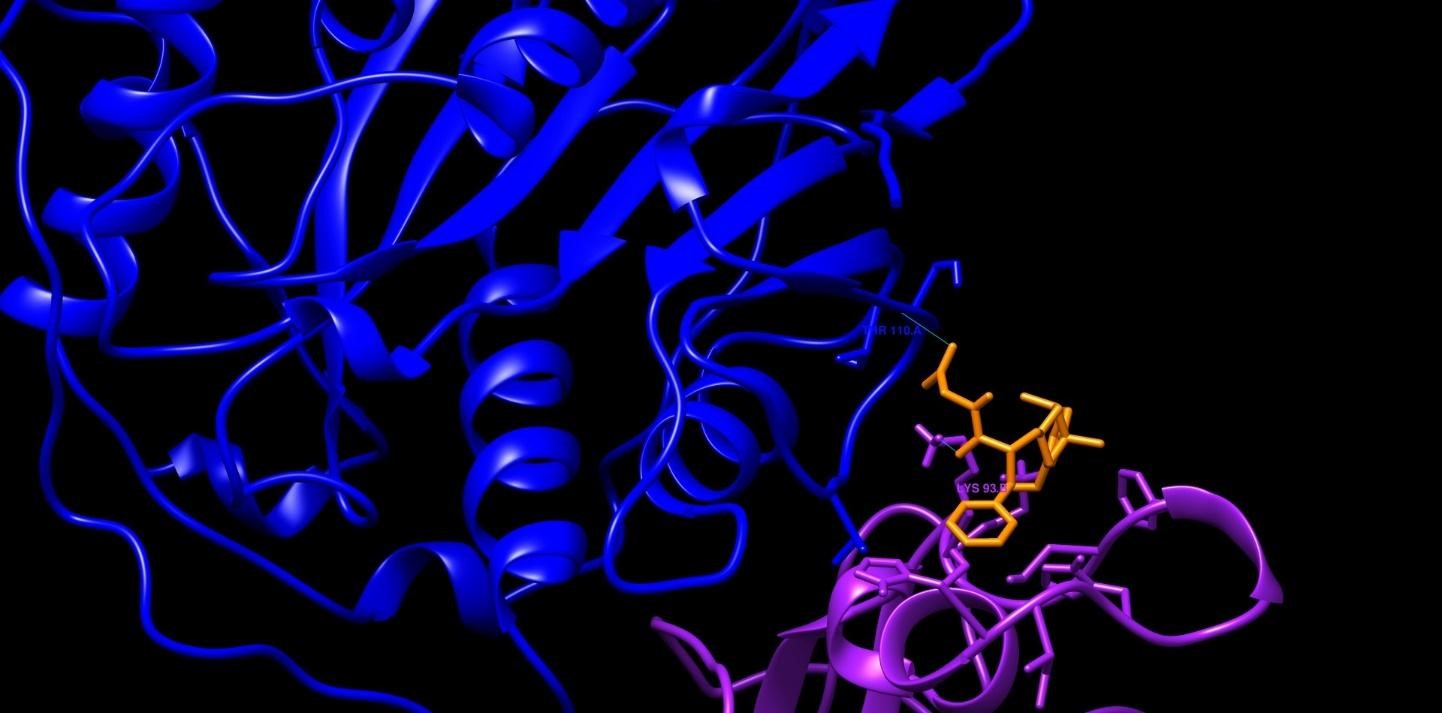

Supplement: S5 Fig — NSP 10–16 complex interaction with Alvimopan. Drugs are in orange while the NSP16 is highlighted in purple and NSP10 is labelled in blue. The residues interacting with the drugs are highlighted in light blue. (TIF) [file pone.0248553.s005.tif]
