# Supplementary material for: A comprehensive SARS-CoV-2 genomic analysis identifies potential targets for drug repurposing
Source: PLoS One. 2021 Mar 18;16(3):e0248553. doi: 10.1371/journal.pone.0248553 (PMC7971693; doi:10.1371/journal.pone.0248553)
Supplement: Attachment — Submitted filename: Response to Reviewers.docx [file pone.0248553.s007.docx]

Dear Dr. Malaya Kumar Sahoo,

Academic Editor

PLOS ONE

We thank you, and the reviewers, for the detailed review, and the comments on our manuscript [PONE-D-20-34651] - [EMID:e297d3cb56d8414a] entitled “A Comprehensive SARS-CoV-2 Genomic Analysis Identifies Potential Targets for Drug Repurposing”. We have gone through all the comments carefully, and have addressed all the points that were raised by the reviewers in detail. We have modified the paper in response to the remarks, and hope that this revised version of the manuscript is suitable for publication in the journal.

We have appended a detailed response in the following pages.

We look forward to hearing from you.

Thank you.

Best wishes,

Arpit Kumar Pradhan

LMU Munich

Ashwin K. Jainarayanan

University of Oxford

## **Response to Reviewers’ comments:**

**Reviewer #1:** In this Manuscript Anand et al. provide the important findings such as identification of conserved genes and the drugs candidates to target the conserved genes. This approach of identifying stable genes and targeting stable genes with drugs would provide a better therapeutic approach and possible hope in the successive clinical trials.

The manuscript was well written, the experiments well designed, and the conclusions are appropriate. I believe that the findings of the manuscript are of sufficient novelty and breadth to merit publication in PLOS ONE.

Below are suggestions for minor revision of the manuscript:

1. Sentences are too long in the abstract section, I would like to recommend split the sentences in abstract.

**Response 1:**

The sentences in the abstract have been rephrased.

One of the recent studies suggests the existence of three central variants of SARS-CoV-2 distinguished by amino acid changes 13. We urgently need therapeutic options to combat this virus infection.

2. The sentences above are important for the introduction part but it would sound better, if the authors can bring some coherence.

**Response 2:**

We have rewritten the introduction to address this. Please refer to the track change version to infer the exact changes.

3. “This approach of targeting the stable genes for drug discovery would provide a better therapeutic approach and confidence in the successive clinical trials”.

Please check the bioavailability of the identified drugs (in plasma and organ tissues) whether the drug candidates has possible therapeutic efficacy at MTD drug doses.

**Response 3:**

We have introduced a new paragraph under the headline of “Side-Effects of the drugs chosen for targeting” in the results and discussion section providing detailed information about the bioavailability and MTD dosage for all the repurposed drugs suggested in the manuscript. The references for the respective drugs are added in the bibliography section.

However I have no major concerns with this paper in its current form.

**Reviewer #2**: In the presented manuscript, Anand et al. authors performed a phylogenetic analysis of SASR-COV-2 variants to determine the most conserved region of the virus and performed a virtual screening against determined proteins FDA-approved drugs. Further, they performed molecular docking and determined the drugs Darifenacin, Nebivolol, Bictegravir, Alvimopan, and Irbesartan as the most promising candidates.

In the first part of the paper, the phylogenetic analyses are unnecessary and misleading. There is a number of high-quality publications (e.g. doi.org/10.1101/2020.04.17.046086 ., DOI: 10.1002/jmv.25902, DOI: 10.1002/jmv.25699) and publicly available resource for phylogenetic analysis of SARS-COV-2 variants https://nextstrain.org/. Therefore, to avoid controversial and unsupported conclusions of coronavirus's potential origin and transmission roads, I recommend focusing on virtual screening and docking of the FDA-approved drugs, presented in the second part of the manuscript.

With the modifications, the paper could be suitable for publication

**Response:**

We understand the reviewer’s concern. We have removed our phylogenetic analysis from the first part of the manuscript. We have paid more attention towards the virtual screening, docking and MD simulations of the repurposed FDA approved drugs in the manuscript. Instead of 25 ns simulations that were initially done, the MD simulations are now extended to an optimal time of 100 ns. The time of 100 ns was chosen since the RMSD of the protein-drug complexes stabilized in the early phases of the simulation. Respective changes have been made under the “Molecular Dynamic simulations” in the results and discussion section and in the “Materials and Methods” section. The figures for 100 ns MD simulations have been added in the “Molecular Dynamic simulations” section. The various parameters that were considered in the MD analysis for each protein have been listed in a separate table (Table 4).

**Reviewer #3:** This paper presents a very standard approach to both the phylogenetic analysis of SARS-CoV2 and the homology modeling building and virtual ligand screening. There are a number of issues that preclude publication in the following form:

1. The paper is disjoint. What insights does the phylogenetic analysis provide with respect to the drug repurposing aspect of the algorithm?

**Response 1:**

We have removed our phylogenetic analysis from the first part of the manuscript and have edited the manuscript in order to bring coherence to the story. We have assigned more weight to the conserved gene approach and drug targeting in the current manuscript.

2. There are numerous technical and conceptual problems with the ligand screening/homology modeling. 25 ns or so simulations are too short to see significant structural changes. How were the the particular drugs chosen? What is the point of doing a Lipinski analysis on FDA approved drugs?

**Response 2:**

We have increased the simulation timings to 100 ns in order to observe structural details at a finer level. The time of 100 ns was chosen since the RMSD of the protein-drug complexes stabilized in the early phases of the simulation. During the screening of ligands for each protein, the drugs from the FDA approved database were docked to the protein and the PLANTS software provided a docking score for each of the drugs. For every docked protein, the top two drug candidates with higher docking score were chosen and were moved for the further MD analysis. We have removed the Lipinski analysis part on the repurposed drugs.

3. The paper targets nsp10. But nsp10 forms a complex with nsp16 which is likely to be a biologically relevant conformation. How does the dimer affect the putative ligand binding with nsp10? Why not simulate the dimer since there is a crystal structure of the nsp10/nsp16 complex?

**Response 3:**

We agree with the comments raised by the reviewer. We have reanalysed the screening, docking and the MD simulation with NSP10-NSP16 complex by using the crystal structure of the complex available in the PDB database with RCSB PDB ID 7C2I. Indeed there was a change in the ligand-binding pocket when the NSP10-NSP16 complex was considered. While the binding of Alvimopan with the complex was stable, the NSP-Irbesartan complex was not stable as evident from the MD analysis. Since both NSP10 and NSP10-NSP16 complex have different preferred ligand-binding sites, we have also decided to retain the simulations that were originally performed with NSP10. We have modified the figures and provided the results of the new analysis.

4. What is the false positive rate of the docking approach that is used? How confident are the binding predictions?

**Response 4:**

We have used PLANTS approach to perform our docking analysis. The tool provides scores which proxies the success rate for each protein-ligand docking. The confidence of these binding predictions for the dockings done in the manuscript has been added in the “Homology modelling of stable targets and virtual screening of small molecules” in the results and discussion section. The rationale behind using the PLANTS approach is explained in the materials and methods section.

5. The authors need to perform experimental validation that their predictions are correct.

**Response 5:**

In order to validate the results from the haplotype analysis, homology modelling, virtual screening and molecular docking, we have performed ab-initio molecular dynamics simulations. The time duration of production MD was chosen to make sure that all the docked complexes were indeed stable. The RMSD of the docked complexes stabilized well before 40ns. However, we continued to simulate the systems for another 60ns to verify the stability of the docked systems.

In addition to computational experiments, several existing published articles indicate that the selected repurposed drugs are effective in combating the viral infection. Some existing literature evidence supporting our study are listed below:

1. Zhou Y, Hou Y, Shen J, Huang Y, Martin W, Cheng F. Network-based drug repurposing for novel coronavirus 2019-nCoV/SARS-CoV-2. Cell Discovery. 2020;6(1).
2. Kim J, Zhang J, Cha Y, Kolitz S, Funt J, Escalante Chong R et al. Advanced bioinformatics rapidly identifies existing therapeutics for patients with coronavirus disease-2019 (COVID-19). Journal of Translational Medicine. 2020;18(1).
3. Guan W, Lan W, Zhang J, Zhao S, Ou J, Wu X et al. COVID-19: Antiviral Agents, Antibody Development and Traditional Chinese Medicine. Virologica Sinica. 2020;35(6):685-698.
4. Akhtar S, Benter I, Danjuma M, Doi S, Hasan S, Habib A. Pharmacotherapy in COVID-19 patients: a review of ACE2-raising drugs and their clinical safety. Journal of Drug Targeting. 2020;28(7-8):683-699.
5. Chatterjee B, Thakur S. ACE2 as a potential therapeutic target for pandemic COVID-19. RSC Advances. 2020;10(65):39808-39813.
6. Enmozhi S, Raja K, Sebastine I, Joseph J. Andrographolide as a potential inhibitor of SARS-CoV-2 main protease: an in silico approach. Journal of Biomolecular Structure and Dynamics. 2020;:1-7.
7. Bocci G, Bradfute S, Ye C, Garcia M, Parvathareddy J, Reichard W et al. Virtual and In Vitro Antiviral Screening Revive Therapeutic Drugs for COVID-19. ACS Pharmacology & Translational Science. 2020;3(6):1278-1292.

Overall, this paper represents an incremental contribution to a very important and difficult problem.

________________________________________
